# The Role of Acupuncture Improving Cognitive Deficits due to Alzheimer's Disease or Vascular Diseases through Regulating Neuroplasticity

**DOI:** 10.1155/2021/8868447

**Published:** 2021-01-12

**Authors:** Shaozhen Ji, Jiayu Duan, Xiaobing Hou, Li Zhou, Weilan Qin, Huanmin Niu, Shuyun Luo, Yunling Zhang, Piu Chan, Xianglan Jin

**Affiliations:** ^1^Department of Neurology, Dongfang Hospital, Beijing University of Chinese Medicine, Beijing 100078, China; ^2^Department of Neurobiology, Xuanwu Hospital of Capital Medical University, Beijing 100053, China; ^3^National Clinical Research Center for Geriatric Disorders, Capital Medical University, Beijing 100053, China; ^4^Beijing University of Chinese Medicine, Beijing 100029, China; ^5^Department of Neurology, Beijing First Hospital of Integrated Chinese and Western Medicine, Beijing 100039, China; ^6^Department of Acupuncture and Moxibustion, Dongfang Hospital, Beijing University of Chinese Medicine, Beijing 100078, China; ^7^Xiyuan Hospital, China Academy of Chinese Medical Sciences, Beijing 100091, China

## Abstract

Dementia affects millions of elderly worldwide causing remarkable costs to society, but effective treatment is still lacking. Acupuncture is one of the complementary therapies that has been applied to cognitive deficits such as Alzheimer's disease (AD) and vascular cognitive impairment (VCI), while the underlying mechanisms of its therapeutic efficiency remain elusive. Neuroplasticity is defined as the ability of the nervous system to adapt to internal and external environmental changes, which may support some data to clarify mechanisms how acupuncture improves cognitive impairments. This review summarizes the up-to-date and comprehensive information on the effectiveness of acupuncture treatment on neurogenesis and gliogenesis, synaptic plasticity, related regulatory factors, and signaling pathways, as well as brain network connectivity, to lay ground for fully elucidating the potential mechanism of acupuncture on the regulation of neuroplasticity and promoting its clinical application as a complementary therapy for AD and VCI.

## 1. Introduction

As the population ages, the prevalence of dementia is increasing worldwide with an annual incidence of nearly 10 million [1], which leads to threats and challenges to global health and wellbeing. Dementia is characterized as a syndrome with myriad and complex causes, including primary neurologic, neuropsychiatric, and medical conditions and genetic and environmental factors [2, 3]. In the elderly, neurodegenerative dementias are most common [2], among which Alzheimer's disease (AD) is believed to be the leading cause of dementia, and vascular cognitive impairment (VCI) is the second utmost cause [4, 5]. Unprecedented advancements have been made in molecular neuroimaging, clinicopathologic correlation, and the development of novel biomarkers in recent decades. However, effective therapeutics remain limited and even absent to date [4, 5]. Acupuncture, as one of complementary therapies for AD and VCI, is gradually applied to alleviate suffering, aggressively treating contributing symptoms and improving overall quality of life [6–10]. However, the underlying mechanisms remain elusive.

Neuroplasticity refers to the capacity of the nervous system to adapt to internal and external environmental changes by reorganizing its structure, function, and connections [11–14], which occurs at various levels of the nervous system from tissue to cellular to molecular [13]. It is known that dysregulated or disrupted neuroplasticity is implicated as a pathological mechanism in AD [15] and VCI [16]. Furthermore, some treatments that stimulate or modulate neuroplasticity have been indicated as effective in improving cognition [12, 17, 18], and might be potential therapy in cognitive impairments such as AD and VCI.

Acupuncture signals are recognized as a potent form of sensory stimulation that ascend mainly through the spinal ventrolateral funiculus to the brain [19]. The mechanisms of acupuncture-mediated neuroplasticity have recently attracted increased interest. Accordingly, acupuncture modulation over several cognition- or aging-related gene expressions [20], plasticity signaling pathways [21, 22], and brain functional connectivities [23] has been studied. Herein, we review the application of different protocols of acupuncture in animal models and humans, and their effectiveness on neuroplasticity in various sections: neurogenesis and gliogenesis, synaptic plasticity, related proteins and signaling pathways, and brain network connectivity. This review is aimed at laying the ground for elucidating the potential mechanism of acupuncture on AD and VCI to promote its clinical application as a complementary treatment.

## 2. Neurogenesis and Gliogenesis

The proliferation and differentiation of neurons and glial cells, also known as neurogenesis and gliogenesis, contribute to some neurorepair and improve brain function [24, 25]. Many previous results demonstrated that cerebral amyloidosis in AD mouse models caused neuronal proliferation inhibition and marked gliogenesis [26–28], and that stroke could trigger striatal and cortical neurogenesis and gliogenesis in murine models [29]. Mounting evidence indicates that adult hippocampal neurogenesis is implicated in cognitive processes, and that neurogenesis deficits may impair learning and memory. In states of brain injury such as AD and VCI, compensatory neurogenesis and gliogenesis mediate a balance between initial injury processes and endogenous repair processes [24]. Regulation of neurogenesis and gliogenesis is possibly associated with improving cognitive impairment and, consequently, may be attractive therapeutic targets for AD and VCI.

It is known that neurogenesis in the adult mammalian brain mostly takes place in specific brain regions harboring adult neuro stem and precursor cells, such as the subgranular zone (SGZ) of the hippocampal dentate gyrus (DG) and the ventricular/subventricular zone (VZ/SVZ) of the lateral ventricles [25]. Cognitive impairment due to AD or ischemic injury is recognized as partly related with neuron loss, impairment of cell proliferation, and imbalance between neuron loss and proliferation in the above regions [30]. Some studies showed that both manual acupuncture (MA) and electroacupuncture (EA) could ameliorate the learning and memory deficits of AD mice models through inducing the enhancement of neuron proliferation and migration in hippocampal DG and VZ/SVZ [31–33]. And the effect of MA and EA on improving cognitive dysfunction through the proliferation and differentiation of hippocampal neuro stem cells (NSCs) was also identified in murine models for vascular dementia (VaD) [34–36]. In addition, neurogenesis could take place in other brain areas in pathological conditions, such as the cortex [37], where the promotion of neurogenesis related to EA was also detected in the transgenic mice model for AD [33].

VCI is recognized to be associated with pathological changes in white matter degeneration and demyelination [38]. Oligodendrocyte (OL), as one predominant cell type in white matter, mediates myelination that is an essential process for the appropriate propagation of action potentials along axons [39]. Myelination participates in the restoration of damaged white matter in the adult brain [40], which may provide potential utility for the treatment of VCI. In a mouse model of VaD, EA was indicated to enhance the differentiation of oligodendrocyte precursor cells (OPCs) into mature OLs and ameliorate white matter damage in the corpus callosum (CC) [41]. Moreover, astrocytes also perform critical impacts on promoting neovascularization, regulating neuronal activity, and supporting synaptogenesis and neurogenesis, which may influence recovery following ischemic lesion [39, 42]. Experimental studies have reported that acupuncture was able to influence the proliferation and differentiation of astrocytes; however, the results were discrepant. One study revealed that MA was able to inhibit astrocyte activation and proliferation in VaD rat models [36]. Conversely, Kim et al. found that EA stimulation could induce NSCs differentiated into astrocytes in a VaD mouse model [35]. These results may be caused by differential acupoints or acupuncture methods. The differential influence of the acupuncture method (i.e., MA vs. EA) on neurogenesis has been demonstrated. And one study found that MA vs. EA stimulation at the same acupoints might induce differential cell proliferation and neuroblast differentiation in healthy rats [43]. And further investigation of the compared impact of differential acupuncture methods and acupoints on gliogenesis in AD and VCI models is required.

In addition to the direct effect on endogenous neurogenesis and gliogenesis, acupuncture was able to promote the survival, proliferation, migration, and differentiation of exogenous NSCs in the hippocampal microenvironment by regulating components of the cerebral microenvironment [44] or the related cytokine levels [45] in an AD mice model. All these findings demonstrated the influence of acupuncture on endogenous and exogenous neurogenesis and gliogenesis in AD and VCI, which deepen our understanding of acupuncture modulating neuroplasticity. There remain some limitations and even discrepancies in these results possibly caused by acupoints or models or observation times, or even acupuncture methods (i.e., MA vs. EA). And the mechanisms underlying the impact of acupuncture on neurogenesis and gliogenesis in different states, especially molecular mechanisms, need to be investigated.

## 3. Synaptic Plasticity

Synapses, the most sensitive and plastic structures, are directly involved in the integration and transfer of information within the neuro system. Previous studies demonstrated that synapse loss and dysfunction was a key feature in AD [46] and VCI [47] and positively correlates with cognitive damage. Impaired dendritic structure, spine density, and synaptic ultrastructure of neurons have been identified in brain tissue of AD patients and murine models, caused by soluble amyloid beta (A*β*) in the hippocampus [48, 49]. And ischemia-induced synapse reduction was also recognized to be the major pathological causes of VaD [50]. Synaptic plasticity, also defined as activity-dependent synaptic modifications of the strength of synaptic connections, is widely recognized to be fundamental to the formation and maintenance of learning and memory [51]. Synaptic plasticity in the neuro network, an important basis for cortical plasticity, is associated with learning and memory and sensorimotor dysfunction and recovery [51, 52]. Synaptic plasticity mainly includes modulation of the morphological structure of synapses and the synaptic strength and transmission, in which some synaptic protein markers, neurotransmitters, and receptors participate. Recently, modulation of synaptic plasticity is believed to be a promising approach for treating AD and VCI.

Synapse-structure parameters, such as synaptic curvatures, the width of the synaptic cleft, and the thickness of the postsynaptic density, are proposed to be important indicators that reflect synaptic morphological plasticity and greatly affect synaptic transmission [53]. Many studies revealed that MA and EA treatments had positive effects on the recovery of the learning and memory abilities not only in AD rat models but also in VCI, through increasing synaptic curvatures, decreasing the width of synaptic clefts, and thickening the postsynaptic densities in the hippocampus [49, 54]. In addition, MA was able to reverse the learning and memory impairments in AD mice models through enhancing the conjunction among the synapses and promoting synaptic formation [20] and regeneration [55], reducing ultrastructural degradation of synapses [56], and increasing the number and length of dendrites [57] and neurite fibers [58].

Long-term potentiation (LTP) and long-term depression (LTD) are considered as two indicators and forms of synaptic transmission [59]. As a cellular model of synaptic plasticity, LTP is the long-lasting enhancement in signal transmission between two neurons after synchronous stimulation associated with memory formation and storage, reflecting an increase of synaptic strength [60]. LTD is relevant to memory integration, forgetting, and recovery of LTP production at desaturation state [61]. And converging studies supported a crucial role of LTD in some types of learning and memory and in situations where cognitive demands require a flexible response [59]. Many electrophysiological studies showed that acupuncture could apparently improve the recovery from cognitive deficits by promoting LTP and/or LTD [61–63] and preventing or restoring the impaired LTP [64–69] in AD or VCI rat models. In addition to LTP and LTD, EA could also ameliorate the synaptic transmission by raising the slope of excitatory postsynaptic potential (EPSP) and the amplitude of population spikes (PS) in an AD mouse model [70].

Synaptophysin (SYN) is a major integral membrane protein of the presynaptic vesicle, and postsynaptic density 95 (PSD-95) and growth-associated protein 43 (GAP-43) are postsynaptic markers [71]. As important protein markers of regeneration and remodeling, they are widely found in all nerve terminals and used for quantifying the number of axon terminals, reflecting the occurrence, density, and strength of synapses [49, 72]. Many previous studies reported reduced expression of SYN and PSD-95 in the hippocampus in AD and VaD [73, 74]. It was demonstrated that acupuncture was able to promote synapse-structure damage rehabilitation by upregulating the expression of SYN [44, 54, 55], PSD-95 [56, 75, 76], and GAP-43 [77] to improve the learning and memory abilities of AD and VCI murine models.

Furthermore, accumulated evidence indicates that the effect of acupuncture on modulating synaptic structure and function in AD and VCI is achieved by changing the releasing of the presynaptic neurotransmitter or the function of the postsynaptic receptor [67, 68, 78]. As one of the major neurotransmitters, dopamine (D) plays an essential role in modulating hippocampal LTP and memory processes [79, 80]. Ye et al. found that MA could activate D1/D5 receptors to ameliorate cognitive function and LTP impairments in VaD rats [67]. The central cholinergic pathway and the norepinephrine- (NE) adrenergic receptor (AR) system are known for their critical roles in learning acquisition and synaptic plasticity in the mammalian limbic system. It was demonstrated that MA not only could alleviate memory-associated decreases in the levels of choline acetyltransferase (ChAT) and restore the expression of choline transporter 1 (CHT1) as well as vesicular acetylcholine transporter (AChT), resulting eventually in the recovery of the entire cholinergic system circulation pathway [81], but also was able to enhance norepinephrine (NE) levels and the activation of *β*1-AR in the hippocampus [68]. In addition, *γ*-aminobutyric acid (GABA) is one main inhibitory neurotransmitter in the central nervous system inhibiting the excessive release of glutamate (Glu). And GABA receptor-mediated inhibitory inputs modulate hippocampal LTP [82]. EA could elevate the excitability of granule cells by decreasing GABA from interneurons, which resulted in increasing LTP [78].

Glutamate receptors (GluRs) are the main receptors of the postsynaptic neurotransmitter area and modulate synaptic plasticity; they are divided into metabotropic GluRs and ionotropic GluRs. Among the three types of ionotropic GluRs, N-methyl-D-aspartate receptor (NMDAR) is the most widely distributed regulator of synaptic plasticity, which plays an important role in inducing and maintaining LTP and LTD closely associated with learning and memory [83]. NMDARs are comprised of NMDAR subtype 1 (NMDAR1) subunits plus at least one type of NMDAR2 subunit [84]. It was reported that EA could reduce the deficit of LTP in VaD rat models via reversal of NMDAR1- and transient receptor potential vanilloid subtype 1- (TRPV1-) mediated neurotoxicity [62]. NMDAR2 seems to have complex properties, and different NMDAR2 subunits confer distinct electrophysiological and pharmacological properties on the receptors and couple themselves with opposing signaling pathways and influences on the direction of synaptic plasticity [85]. Specifically, NMDAR2A activation is beneficial for neuronal regeneration and neuroprotection, while NMDAR2B induces neurotoxicity and neuronal apoptosis [85]. One study found that EA could alleviate cognitive dysfunction caused by ischemic injury through downregulation of NMDAR2B and upregulation of NMDAR2A [86].

The effect of EA on synaptic plasticity might be related to the parameter of stimulation. One study has found that high-frequency EA may yield a stronger protective effect on hippocampal synaptic plasticity compared with low- or medium-frequency EA in AD rat models [61]. Further research focusing on ascertaining the optimum acupuncture parameter is required. Moreover, besides these mechanisms described above, many synaptic-related proteins or signaling pathways were required in maintaining synaptic structural plasticity and synaptic transmission. Investigations of synaptic plasticity-related regulatory factors and signaling mechanisms have been performed in many studies, and these are going to be described in Section 4.

## 4. Neuroplasticity-Related Regulatory Factors and Signaling Pathways

Multiple crucial steps are involved in the process of neuroplasticity, which include many layers of regulation, composed of both intrinsic and extrinsic mechanisms. For example, there are a number of coordinated cell-intrinsic programs and external signals involved in distinct stages of adult neurogenesis, including proliferation and lineage differentiation of NSCs, migration of neuroblasts, and integration of newborn neurons [87]. Given the important role of related factors and signaling pathways in neuroplasticity, ascertaining acupuncture's effect on them may be vital to understanding the mechanisms of its treatment for AD and VCI.

As one of the morphogens that are critical during embryonic development of the nervous system, Notch is highly conserved and serves as niche signals to regulate the proliferation of adult NSCs [88]. The regeneration of neurons from neural progenitors may be impaired due to the abnormal elevated Notch signal pathway. EA treatment suppressed neuronal apoptosis and improved cognitive impairment in AD rat models possibly via the downregulation of an abnormal elevated Notch signaling pathway [89]. Moreover, EA also was able to enhance hippocampal NSC proliferation in VaD rat models via the activation of the Notch signaling pathway [34].

In addition to the neurotransmitters described above, the survival and synaptic integration of newly born cells are subject to regulation by neurotrophic factors. As a member of the neurotrophic factor family, the BDNF protein is synthesized as pre–pro-BDNF and cleaved intracellularly into a pro-BDNF protein encompassing two domains: the prodomain and the mature BDNF domain [90]. BDNF is actually secreted in the pro- and mature form [91], which had distinct receptors and signaling cascades resulting in opposing biological functions [92–94]. The mature BDNF preferentially binds to phosphorylated tropomyosin receptor kinase B (Trk-B) receptors leading to cell survival and differentiation as well as hippocampal LTP, whereas pro-BDNF preferentially binds to p75 neurotrophin receptor (p75NTR) leading to apoptosis and hippocampal LTD [95]. It was observed that acupuncture could upregulate the expression of Trk-B receptors and could decrease the expression level of p75NTR in AD and VaD murine models, influence the modulation and processing of the BDNF protein from pro-BDNF to mature BDNF [33, 96, 97], and eventually enhance the mRNA expression levels of mature BDNF [35, 45, 54, 81]. One clinical trial showed that combined scalp acupuncture and cognitive training could improve the cognitive function and BDNF levels of peripheral blood in patients with stroke during the recovery stage [98]. Other extrinsic factors such neurotrophin 3 (NT3), NT4, and NT5 also play an important role in the regulation of neuronal integration [99]. EA treatment has been reported to increase the expression of NT4/5 and their receptor, tyrosine receptor Trk-B, and promote OL regeneration in association with cognitive functional improvements in a VaD mice model [41]. In addition, acupuncture also could regulate intrinsic factors associated with neuronal integration. For instance, MA was demonstrated to restore the expression of cAMP-response element-binding protein (CREB) mRNA in the hippocampus of rats with cognitive impairment [81].

The typical pathological hallmarks of AD include extracellular A*β* plaques and intracellular neurofibrillary tangles (NFTs) composed of hyperphosphorylated tau proteins, both of which resulted in the loss and morphological changes of dendritic spines, directly leading to the damage of neuronal synaptic function and neuroplasticity [100]. Many studies showed that acupuncture could regulate neuroplasticity by directly reducing A*β* deposition [56, 101], and some related proteins and signaling pathways participated in this process. Glycogen synthase kinase 3 beta (GSK3*β*) is a serine/threonine protein kinase that plays a crucial role in AD pathogenesis, and its hyperactivity or overexpression is increasingly shown to be closely related to A*β* generation, tau hyperphosphorylation, and synaptic plasticity [102]. Inhibition of GSK3*β* has been indicated to increase the number of synapses and postsynaptic density thickness, and rescue the reduction of spine density in the hippocampus of an AD model. It has been revealed that EA could promote synapse-structure damage rehabilitation by downregulating GSK3*β* to improve the learning and memory abilities of AD rat models [49, 77]. As the downstream target of GSK3*β*, the reactivation of mTOR restored the acidification of the autophagy lysosome, further promoting the autophagy clearance of pathological A*β* plaque load [103]. Yu et al. found that EA rescued structural and functional synaptic plasticity impairments and memory deficits in AD rat models through the inactivation of GSK3*β*/mTOR signaling [21]. Moreover, *β*-site amyloid precursor protein cleaving enzyme 1 (BACE1) is the key protein involved in A*β* peptide generation. One study indicated that EA could downregulate the expression of BACE1 in one AD mouse model [64].

There are some regulated factors and signaling pathways directly involved in the modulation of LTP. Protein kinase A (PKA) is a predominantly positive modulator of LTP in the hippocampus and has been demonstrated to indispensably participate in the efficacy of hippocampus-based memory [104]. Tang et al. found that EA could upregulate PKA activation, enhance synaptic plasticity, and improve memory in an AD mice model [64]. The p70 ribosomal protein S6 (p70S6) kinase/ribosomal protein S6 signaling pathway has been shown to promote neuronal growth and LTP [105, 106]. One study showed reduced expression of p70S6 kinase and ribosomal protein S6 in the hippocampus of VaD rats, which suggested that the p70S6 kinase/ribosomal protein S6 pathway was involved in the pathogenesis of VaD [63]. EA was demonstrated to improve the learning and spatial memory abilities of VaD rats and facilitate LTP in the hippocampus by upregulating expression of p70S6 kinase and ribosomal protein S6 [63]. The p70S6 kinase was phosphorylated by activation of the mammalian target of rapamycin (mTOR) signal pathway, which has been shown to promote neuronal growth and LTP [107, 108]. Acupuncture stimulation has been indicated to promote neuroplasticity by regulating the mTOR signal pathway in AD or VaD rats [21, 109]. Moreover, it was reported that MA could reverse the learning and memory impairments in an AD mouse model through upregulating eukaryotic Y-box-binding protein (YB-1) expression [20], which enhanced the conjunction among the synapses and promoted synaptic formation indirectly [110]. The eukaryotic elongation factor-2 kinase/eukaryotic elongation factor-2 (eEF2K/eEF2) pathway is also associated with synaptic plasticity and its inhibition prevents synaptic failure in AD. One study showed that EA improved the synaptic function in AD by inhibiting the AMPK/eEF2K/eEF2 pathway in an AD mouse model [76].

**Figure 1:**
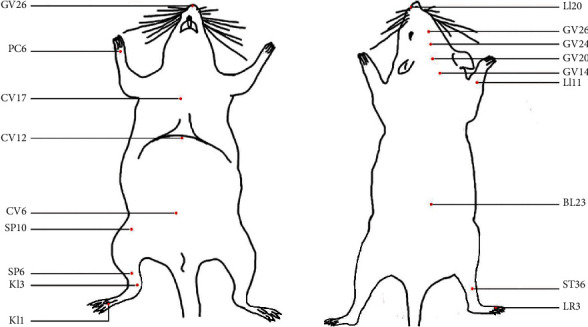
The locations of acupoints in mice.

Besides the above-related factors and signaling pathways, other mechanisms, such as oxidative stress, glucose metabolism, and inflammatory responses, were considered to play a key role in acupuncture treating AD or VCI and modulating neuroplasticity (Table 1 and Figure 1). These molecular mechanisms support acupuncture as a potentially promising therapy for the treatment of cognitive dysfunction in patients with VD or VCI.

## 5. Brain Network Connectivity

Some previous neuroimaging researches have revealed neuropathological changes and/or structural-functional reorganization in AD and VCI resulting in altered connectivity patterns in brain networks [14, 111–113]. For example, some rapidly and reversibly increased or decreased strengths of brain network connections, also known as altered recruitments of functional connections normally devoted to performing a given task or the recruitment of additional network connections that are not typically activated by healthy people. And the alteration of network connectivity is a form of neuroplasticity that could indicate compensatory mechanisms engaged to maintain a normal level of cognitive function or promote the recovery from cognitive dysfunction due to the primary white matter lesions and neuronal loss [14, 114, 115].

**Figure 2:**
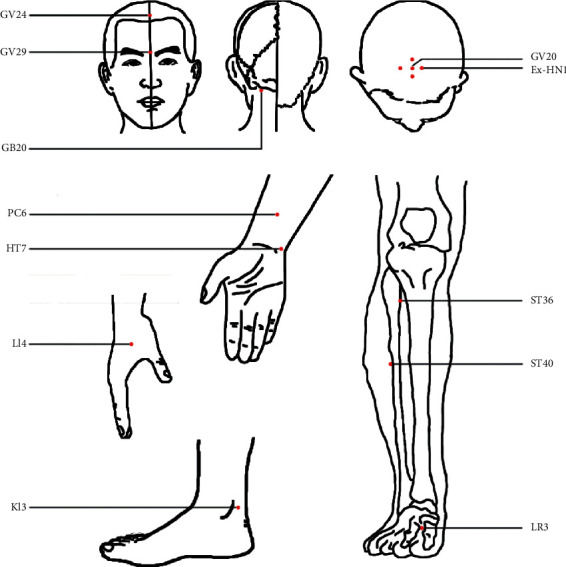
The locations of acupoints in humans.

Many neuroimaging studies showed that acupuncture could induce neuroplastic reorganization of brain functional networks in AD or mild cognitive impairment (MCI), the prophase state of AD [116] (Table 2 and Figure 2). There were several regions showing increased or decreased activities in MCI and AD patients after short-term MA or EA stimulation, including cognitive-related areas, visual-related areas, sensorimotor-related areas, emotion-related areas, the basal ganglia, and the cerebellum [23, 113, 117–123]. However, there remains a lack of correlation between the changes in cognitive function and alteration in functional connectivity. In two other long-term studies, MCI patients exhibited improvement of cognitive performance after MA, as well as extensive activation and deactivation in brain networks [123, 124]. And functional connectivity strength in some regions was negatively correlated with the changes in memory scores [125], which offered evidence in support of compensatory mechanisms triggered to overcome cognitive deficits in MCI. These findings might provide a deep understanding of acupuncture's therapeutic effect in AD.

Acupuncture's influence on brain network connectivity might be correlated to acupoints, depth of stimulation, and frequency of EA stimulation in AD and MCI. The synergistic effects of different single acupoints or combined acupoints could activate different brain areas and impact the therapeutic effects of acupuncture [116]. And deep stimulation at appropriate acupoints could perform stronger or more extensive effective connectivity related to the therapeutic effect compared with superficial stimulation [119, 121, 126]. Furthermore, high-frequency EA may induce more specific targeted brain response or strengthen the functional connectivity of brain networks associated with memory and cognition. Thus, the impact of acupoint specificity, needling depth specificity, and EA parameter specificity on brain network connectivity in future neuroimaging studies also needs to be elucidated. Since few fMRI imaging studies have been reported regarding acupuncture in patients with VCI, the effect of acupuncture on neuroplastic reorganization of brain functional networks in VCI is still to be established.

## 6. Discussion

**Figure 3:**
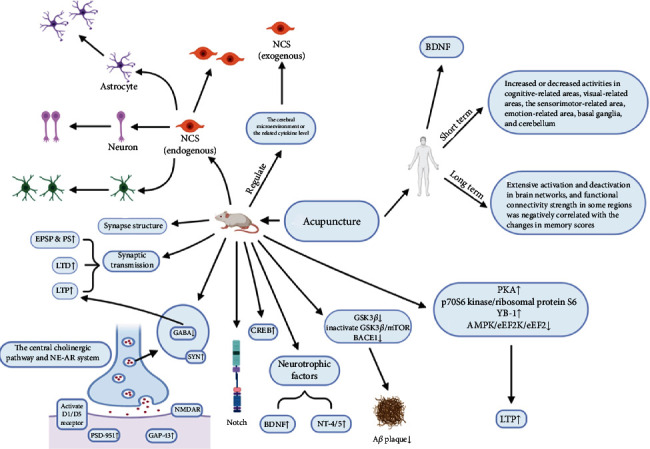
Mechanisms involved in acupuncture regulating neuroplasticity to improve cognitive function.

In addition to directly attenuating the deposition, neuroinflammatory response, and neurotoxicity of A*β* [127] and increasing cerebral blood flow [128], acupuncture also could improve cognitive abilities through regulation of neuroplasticity (Figure 3). The improvement of the cellular/molecular microenvironment and recruitment of unaffected or additional brain networks might play important roles in this process. For example, the modulation of the neurotransmitter system involved in the improvement of the cellular/molecular microenvironment may be another candidate potential mechanism through which acupuncture could regulate neuroplasticity [44]. Moreover, it has been demonstrated that other methods in popular practice could increase cognitive reserve and resilience by regulating neuroplasticity, e.g., physical exercises, stimulating psychosocial experiences, meditation, mind games/puzzles, or dietary changes. It will be interesting to investigate whether acupuncture could increase cognitive reserve and resilience in the elderly. And the results would greatly expand the clinical application of acupuncture. Furthermore, identification of differential impacts of manipulation on brain networks may contribute to understanding the mechanisms of acupuncture in neuroplasticity. The comparison between acupuncture and sham/placebo acupuncture occurred in few clinical studies [124], which indicated increased connections between cognition-related regions by acupuncture not sham/placebo acupuncture. In the further researches, diffusion tensor imaging (DTI) of white matter microstructure adjacent to the primary somatosensory cortex and magnetic resonance spectroscopy (MRS) would be used to explore potential differential mechanisms of manipulation.

There are still some inevitable limitations in this review. First of all, because of differences in the quality of included animal studies, such as sample size calculations, experimental animals and procedures, housing and husbandry conditions, intervention, and assessment of experimental outcomes, the heterogeneities cannot be totally avoided (Supplementary Table 1). Second, it is well known that the efficacy of acupuncture stimulation was partly defined by the characteristic sensation “de qi” clinically (a composite of sensations including soreness, numbness, distention, heaviness, and other sensations) [129]. The efficacy of interventions could not be estimated in animal studies. Third, there are differential influences on neuroplasticity due to acupuncture manipulation. For instance, experimental outcomes may be differently attributed to intervention performed by the acupuncture method (i.e., MA vs. EA) and acupoints [35, 36]. Since the number of studies was small, some pathways affected by the manipulation of acupuncture were not discussed, for instance, synaptophysin expression, modulation of neurotransmitter, and neuroplastic reorganization of brain functional networks (Supplementary Table 2).

## 7. Conclusion

A growing number of contemporary studies have gradually validated acupuncture's traditional uses in treating AD and VCI. In view of acupuncture's therapeutic efficiency and regulation of neuroplasticity, it may be beneficial to develop acupuncture as a potentially promising therapy for AD and VCI. However, the exact mechanisms underlying acupuncture's influence on neuroplasticity is still unknown. In addition, identification of differential impacts of acupoint specificity, acupuncture method specificity, depth specificity, cognitive state specificity, and EA parameter specificity on neuroplasticity may contribute to understanding the mechanisms of acupuncture in AD and VCI. These may be important future challenges in standardized clinical applications.

## Figures and Tables

**Table 1:** The mechanisms of acupuncture regulating neurogenesis, gliogenesis, and synaptic plasticity.

Acupuncture intervention			Model/participants	Mechanisms	Refs.
	Acupoint	EA parameters			
		Intensity, frequency			
MA	Qihai (CV6), Zhongwan (CV12), Danzhong (CV17), bilateral Xuehai (SP10), bilateral Zusanli (ST36)	—	SAMP 8 mice	Induce different cell proliferation in different brain regions, and increase neuron density in hippocampal CA3 and DG. Promote the proliferation, migration, and differentiation of exogenous neural stem cells via increasing SYN mRNA and protein levels. Increase the number of apical and basal dendritic branches and total length of apical and basal dendrites. Improve the distribution and arrangement of nerve fibers.	[31, 32, 44, 57, 58]
MA	Bilateral Zusanli (ST36)	—	Multi-infarction dementia modeled in rats with 3% microemboli saline suspension injected into the internal carotid artery	Increase the number of pyramidal neurons, and tend to decrease the number of astrocytes in the hippocampal CA1 area.	[36]
EA	Baihui (GV20)	1 mA, 2/15 Hz	APP/PS1 transgenic mice	Attenuate A*β* deposits, upregulate the expression of BDNF, and promote neurogenesis in both the hippocampus and cortex.	[33]
EA	Baihui (GV20), Dazhui (GV14)	2 V, 2 Hz	MCAO mice	Increase the number of proliferative cells and differentiated cells in the hippocampus and SVZ of the ipsilateral hemisphere, promote differentiation of proliferated cells into neurons or astrocytes, and upregulate mRNA expression of BDNF and VEGF.	[35]
EA	Bilateral Quchi (LI11), bilateral Zusanli (ST36)	NA, 1 or 20 Hz	MCAO rats	Activate the crucial signaling molecules in the notch signaling pathway, increase the secretion of BDNF and GDNF, and promote the proliferation of hippocampal NSCs.	[34]
EA	Baihui (GV20), Dazhui (GV14)	2 V, 2 Hz	BCAS mice	Promote the differentiation of OPCs into OLs, and mediate positive changes in the expression of NT4/5 and its receptor Trk-B.	[41]
MA	Qihai (CV6), Zhongwan (CV12), Danzhong (CV17), bilateral Xuehai (SP10), bilateral Zusanli (ST36)	—	SAMP 8, coculture model of hippocampal tissue specimens, and NSCs in vitro	Increase the count of NeuN- and GFAP-positive cells, regulate the cytokine levels associated with survival, proliferation, and differentiation of NSCs (upregulate the expression of basic FGF, EGF, and BDNF).	[45]
MA	Qihai (CV6), Zhongwan (CV12), Danzhong (CV17), bilateral Xuehai (SP10), bilateral Zusanli (ST36)	—	SAMP 10 mice	Enhance the conjunction among the synapses and hasten the new synapse formation by upregulating YB-1 expression.	[20]
EA	Baihui (GV20), Shenshu (BL23)	NA, 2, 30 and 50 Hz	Rat models of AD induced by injecting A*β*1-42 into the bilateral lateral ventricles	Increase synaptic curvatures, decrease the width of synaptic clefts, thicken postsynaptic densities, and downregulate the expression of GSK3*β*, amyloid precursor protein, and A*β*1-40. Promote synaptic damage rehabilitation by downregulating GSK3*β* and upregulating GAP-43.	[49, 77]
MA+EA	Yintang (GV29), bilateral Yingxiang (LI20)	1.5 mA, 15 Hz (bilateral LI20)	SAMP 8 mice	Inhibit the phosphorylation of p38MAPK and the excessive activation of MG in the hippocampus to reduce the neuroinflammatory response and neurotoxicity of A*β* and promote synaptic regeneration.	[55]
EA	Bilateral Taixi (KI3)	1 mA, 2 Hz	5XFAD mice	Alleviate neuroinflammation, reduce ultrastructural degradation of synapses via upregulation of SYN and PSD-95 protein, and decrease MG-mediated A*β* deposition.	[56]
MA	Bilateral Taixi (KI3), bilateral Taichong (LR3)	—	MCAO rats	Promote the expression of BDNF and SYN, and synaptic structure reconstruction by increasing the postsynaptic density, narrowing the synapse cleft width.	[54]
EA	Baihui (GV20), Shenshu (BL23)	NA, 2 or 50 Hz	Rat models of AD induced by injecting A*β*1-42 into the bilateral lateral ventricles	Increase the ranges of LTP and LTD.	[61]
EA	Baihui (GV20), Yintang (GV29), Shuigou (GV26)	1 mA, 1 Hz	APP/PS1 transgenic mice	Reduce BACE1 deposition and regulate PKA and LTP.	[64]
EA	Baihui (GV20), Dazhui (GV14), bilateral Shenshu (BL23), bilateral Yongquan (KI1)	1-2 mA, 4 Hz	AD model rats established by injecting A*β*25-35 into the bilateral dentate gyri of the hippocampal CA1 area	Raise the slope of EPSP and the amplitude of PS.	[70]
EA	Baihui (GV20), bilateral Zusanli (ST36)	NA, 2 Hz	Diabetes mellitus and cerebral ischemia model rats	Restore impaired LTP.	[65]
EA	Baihui (GV20)	2 mA, 2 Hz	MACO rats	Reduce the deficit of LTP via reversal of NMDAR1- and TRPV1-mediated neurotoxicity.	[62]
EA	Baihui (GV20), Dazhui (GV14), bilateral Shenshu (BL23)	4 mA, 2 Hz	MACO rats	Promote LTP and upregulate expression of p70S6 kinase and ribosomal protein S6 in the hippocampus.	[63]
MA	Baihui (GV20), Zusanli (ST36)	—	2VO rats by bilateral common carotid artery occlusion	Decrease ROS production, increase neural cell survival, and improve LTP. Upregulate DBH expression in hippocampus. Prevent impairments of LTP, promote the release of dopamine and its major metabolites in the hippocampus, and decrease D1 receptors and D5 receptors in the hippocampal DG region. Enhance LTP and NE levels and increase *β*1-ARs in the hippocampus.	[66–69]
EA	Baihui (GV20), bilateral Yongquan (KI1)	0.5 mA, 1/50 Hz	APP/PS1 transgenic mice	Reduce the expression of A*β* in the hippocampus and increase the expression of PSD-95 and SYN.	[75]
EA	Baihui (GV20), Dazhui (GV14), Shenshu (BL23)	1 mA, 2 Hz	SAMP 8 mice	Increase the expression of SYN and PSD-95, and inhibit AMPK activation and eEF2K activity.	[76]
EA	Zusanli (ST36), Sanyinjiao (SP6)	2 mV, 2 Hz	Anesthetized rats	Elevate excitability of granule cells by decreasing GABA from interneurons, which result in increasing LTP.	[78]
MA	Baihui (GV20)	—	Memory defects rats caused by SCO administration	Restore the expression of CHT1, vesicular AChT, BDNF and CREB mRNA in the hippocampus.	[81]
EA	Baihui (GV20), Shenting (GV24)	NA, 1-20 Hz	MCAO rats	Reduce Ca^2+^ influx via inhibition of Glu neurotoxicity and downregulation of NMDAR2B expression.	[86]
EA	Baihui (GV20)	NA, 1 and 20 Hz	APP/PS1 transgenic mice	Enhance the expression levels of mature BDNF and pro-BDNF, and BDNF/pro-BDNF ratio, upregulate the expression levels of phosphorylated Trk-B, and decrease the expression level of p75NTR. Upregulate NAA, Glu and mI metabolism, increase the surviving neurons in the hippocampus, and promote the expression of BDNF and Trk-B.	[96, 97]
MA	Baihui (GV20), Sishencong (EX-HN1), bilateral Fengchi (GB20), Shenting (GV24)	—	Patients with PSCI	Improve BDNF and NGF levels in peripheral blood.	[98]
EA	Baihui (GV20), Shenshu (BL23)	1 mA, 50 Hz	D-galactose-induced aged rats	Attenuate the hippocampal loss of dendritic spines, ameliorate neuronal microtubule injuries, increase the expressions of postsynaptic PSD-95 and presynaptic SYN, and inhibit the GSK3*β*/mTOR signaling pathway.	[21]
EA	Baihui (GV20), bilateral Shenshu (BL23), bilateral Neiguan (PC6), bilateral Zusanli (ST36), bilateral Sanyinjiao (SP6)	NA, 5 Hz	Al/D-gal-OLETF rats	Increase the protein level of p-GSK3*β*.	[22]
EA	Baihui (GV20), Dazhui (GV14), bilateral Shenshu (BL23)	2 mA, 4 Hz	2VO rats by bilateral common carotid artery occlusion	Upregulate expression of mTOR and eIF4E in the hippocampus.	[109]
EA	Baihui (GV20), Shenshu (BL23)	≤2 mA, 20 Hz	Intrahippocampally injected A*β*1–40 rat model	Alleviate the cell apoptosis resulting from A*β* infusion in hippocampal CA1 regions through upregulating the expression of Bcl-2 and downregulating the expression of Bax, promote the expression of synapsin-1 and SYN, and downregulate the level of Notch1 and Hes1 mRNA in the hippocampus.	[89]
MA	Baihui (GV20), Yintang (GV29), Shuigou (GV26)	—	SAMP 8 mice	Improve the level of glucose metabolism in the brain, and the content of A*β* amyloid in the cortex.	[101]

NA: not available. Abbreviations are found in Supplementary Table 3. The locations of acupoints are shown in Figure 1.

**Table 2:** The mechanisms of acupuncture modulating brain network connectivity.

Acupuncture intervention			Participants	Mechanisms	Refs.
	Acupoint	Intervention parameters			
MA	Bilateral Taichong (LR3), bilateral Hegu (LI4)	3 minutes	AD patients vs. HCs	Modulate DMN activity in AD patients, with increased cluster in the left PCC, the right MTG, together with the right IPL and decreased bilateral CG and left PCu within DMN connectivity. Enhance the functional connectivity between the hippocampus and the precentral gyrus in AD patients.	[23, 117]
MA	Bilateral Taichong (LR3), bilateral Hegu (LI4)	3 minutes	MCI patients vs. AD patients vs. HCs	Induce increased or decreased activities in regions of MCI, AD subjects, most of which were involved in the temporal lobe and the frontal lobe closely related to memory and cognition. Induce similar activations in cognitive-related brain areas (inferior frontal gyrus, supramarginal gyrus, and rolandic operculum) as well as deactivations in cognitive-related areas, visual-related areas, basal ganglia, and cerebellum in AD and MCI patients, which were not found in HCs.	[113, 118]
MA	Bilateral Taichong (LR3), bilateral Hegu (LI4)	A total of 4 courses in 6 months (3 times a week, 4 weeks as a course)	MCI patients vs. HCs	Enhance hippocampal FC with ITG and MTG in aMCI subjects.	[125]
MA	Taixi (KI3) on the right side	3 minutes (deep acupuncture vs. superficial acupuncture)	MCI patients vs. HCs	Enhance the correlations related with the temporal regions in MCI patients. Increase the correlations related with the temporal regions for the deep acupuncture condition, compared to superficial acupuncture. Deep acupuncture induces the strongest and most extensive effective connectivities related to the therapeutic effect of acupuncture in MCI patients. Activate 20 brain regions in both MCI and HC participants, including the bilateral anterior cingulate gyrus (BA 32, 24), left medial frontal cortex (BA 9, 10, 11), left cuneus (BA 19), left middle frontal gyrus (BA 11), left lingual gyrus (BA 18), right medial frontal gyrus (BA 11), bilateral inferior frontal gyrus (BA 47), left superior frontal gyrus (BA 11), right cuneus (BA 19, 18), right superior temporal gyrus (BA 38), left subcallosal gyrus (BA 47), bilateral precuneus (BA 19), right medial frontal gyrus (BA 10), right superior frontal (BA 11), left cingulate gyrus (BA 32), left precentral gyrus (BA 6), and right fusiform gyrus (BA 19).	[119–121]
MA	Sishencong (EX-HN1), Yintang (GV29), Neiguan (PC6), Taixi (KI3), Fenglong (ST40), Taichong (LR3)	5 times a week, 4 consecutive weeks (acupoint acupuncture vs. sham acupoint acupuncture)	MCI patients	Increase the connections between cognition-related regions such as the insula, dorsolateral prefrontal cortex, hippocampus, thalamus, inferior parietal lobule, and anterior cingulate cortex.	[124]
EA	Neiguan (PC6) on right side	6 minutes, 1 Hz	AD patients vs. HCs	Activate functions of different brain regions in the HC vs. AD patients, including activation of the frontal lobe, the temporal lobe, and the cingulate gyrus as well as the cerebellum in AD patients, and the frontal lobe and the temporal lobe activated in HCs.	[122]
MA	Shenmen (HT 7), Zusanli (ST36), Fenglong (ST 40) Taixi (KI 3)	NA	AD patients	Induce right main hemisphere activations (temporal lobe, such as hippocampal gyrus, insula, and some area of parietal lobe) and left activated regions (temporal lobe, parietal lobule, some regions of cerebellum)	[123]

NA: not available. Abbreviations are found in Supplementary Table 3. The locations of acupoints are shown in Figure 2.

## Data Availability

Previously reported data were used to support this study and are cited at relevant places within the text as references.

## Supplementary Materials

Supplementary Materials We describe characteristics of included animal studies in Supplementary Table 1 referring to the new ARRIVE guidelines, as well as characteristics of included human studies in Supplementary Table 2. Some abbreviations included in this review are listed in Supplementary Table 3.
