# Supplementary material for: The Role of Acupuncture Improving Cognitive Deficits due to Alzheimer's Disease or Vascular Diseases through Regulating Neuroplasticity
Source: Neural Plast. 2021 Jan 12;2021:8868447. doi: 10.1155/2021/8868447 (PMC7815402; doi:10.1155/2021/8868447)
Supplement: Supplementary Materials — We describe characteristics of included animal studies in Supplementary Table 1 referring to the new ARRIVE guidelines, as well as characteristics of included human studies in Supplementary Table 2. Some abbreviations included in this review are listed in Supplementary Table 3. [file 8868447.f1.docx]

We described characteristics of included animal studies in the Supplementary Table 1 referring to the new ARRIVE guidelines, as well as characteristics of included human studies in Supplementary Table 2.

Some abbreviations included in this review are listed in the Supplementary Table 3.

Supplementary Table 1 Characteristics of included animal studies referring to the new ARRIVE guidelines.

| Sample size | Stimulation parameter | Treatment course | Housing and husbandry conditions | Assessment of experimental outcomes | Refs. |
| --- | --- | --- | --- | --- | --- |
|  |  |  |  |  |  |
|  |  |  |  |  |  |
| 40 (30 SAMP10 and 10 SAMR1) | Manipulation methods for 30 secs. Needle for 30 s with reinforcing method at each point except Xuehai (SP10), which was needled for 30 secs with reducing method. | Once a day for 15 days (no treatment on the eighth day) | According to the National Institute of Health Guide for the Care and Use of Laboratory Animals (NIH Publications No. 80-23). | Gene expression profiling, Northern blotting, RT-PCR | [20] |
| 48 SD | / | 6 times a week, over the course of 8 weeks | Housed at 20 ± 2°C and humidity of 50 ± 10%, and allowed free access to food and water in a 12-hour light/dark cycle. | MWM, Western blots, TEM, Golgi staining | [21] |
| 16 | / | 3 weeks | Housed at 20 ± 2°C and humidity of 60 ± 10%, and allowed free access to food and water in a 12-hour light/dark cycle. | MWM, H&E staining, ELISA, Western blots, RT-PCR | [22] |
| 60 (45 SAMP8 and 15 SAMR1) | Needles were rotated at the rate of twice per second for 30 secs at each point. | Once a day for 15 days (no treatment on the eighth day) | According to the National Institute of Health Guide for the Care and Use of Laboratory Animals (NIH Publications No. 80-23). | MWM, histochemical staining, Stereological evaluation | [31] |
| 60 (45 SAMP8 and 15 SAMR1) | Needles were rotated at the rate of twice per second for 30 secs at each point. | Once a day for 15 days (no treatment on the eighth day) | According to the National Institute of Health Guide for the Care and Use of Laboratory Animals (NIH Publications No. 80-23). | MWM, IHC staining, BrdU staining | [32] |
| NA | / | 20 days | According to the Guidelines for Animal Experimentation of the Fourth Military Medical University and to the National Institute of Health Guide for the Care and Use of Laboratory Animals (NIH Publications No. 80-23). | Immunofluorescence staining, Western blots, Quantification of Aβ1-42 level by ELISA, BrdU staining | [33] |
| 72 SD | / | 6 days | According to the National Institute of Health Guide for the Care and Use of Laboratory Animals (NIH Publications No. 80-23). | Neurological assessment, the infarct volume, IHC staining, Western blots, , Quantification of BDNF and GDNF by ELISA, RT-PCR | [34] |
| 12 | / | 10 days | According to the National Institute of Health Guide for the Care and Use of Laboratory Animals (NIH Publications No. 80-23). | MWM, IHC staining, Western blots, BrdU staining, RT-PCR | [35] |
| 70 Wistar | Needles were rotated at the rate of twice per second for 30 secs at each point. | 14 days (1day rest after 6 days treatment) | According to the Provisions and General Recommendations of Chinese Experimental Animal Administration Legislation. | MWM, Histochemical and IHC staining | [36] |
| 28 C57BL/6 | / | 7 days | According to the National Institute of Health Guide for the Care and Use of Laboratory Animals (NIH Publications No. 80-23). | MWM, Fluoromyelin staining, IHC staining, Western blots, Gene expression profiling, RT-PCR, Trk-B antagonist studies | [41] |
| 60 (50 SAMP8 and 10 SAMR1) | Manipulation methods for 30 seconds. CV17, CV12, CV6, and bilateral ST36 were needled by twisting reinforcing methods, while bilateral SP10 was needled by the twisting reducing method. | Once a day for 15 days (no treatment on the eighth day) | According to the National Institute of Health Guide for the Care and Use of Laboratory Animals (NIH Publications No. 80-23). | MWM, Western blots, RT-PCR | [44] |
| 60 (50 SAMP8 and 10 SAMR1) | Manipulation methods for 30 seconds. CV17, CV12, CV6, and bilateral ST36 were needled by twisting reinforcing methods, while bilateral SP10 was needled by the twisting reducing method. | Once a day for 15 days (no treatment on the eighth day) | According to the National Institute of Health Guide for the Care and Use of Laboratory Animals (NIH Publications No. 80-23). | MWM, Western blots, RT-PCR | [45] |
| 56 Wistar | / | Once a day for 15 days (no treatment on the eighth day) | Housed at 20 ± 2°C and humidity of 55 ± 10%, and allowed free access to food and water in a 12-hour light/dark cycle. | MWM, Western blots, TEM | [49] |
| 204 SD | 30 min/day | 14 days | Housed at 22 ± 1°C and humidity of 55 - 75%, and allowed free access to food and water in a 12-hour light/dark cycle. | mNSS, MWM, IHC staining, Electron Tomography | [54] |
| 40 (30 SAMP10 and 10 SAMR1) | / | 6 days a week for 4 weeks | Housed at 22 ± 2°C and humidity of 55%, and allowed free access to food and water in a 12-hour light/dark cycle. | MWM, Immunofluorescence staining, Western blots, Morphological Assessment and Cell Counts | [55] |
| NA | / | 3 times per week for 2 weeks | According to the animal care guidelines of the Korea Institute of Oriental Medicine. | NOR, Y-maze test, Western blots, Electrophysiology, Quantification of Aβ1-42 level by ELISA, IHC and immunofluorescence staining, MicroPET scanning, TEM | [56] |
| 30 (20 SAMP8 and 10 SAMR1) | Manipulation methods for 30 seconds at each point. | Once a day for 14 days (no treatment on the seventh day) | Housed at 23 ± 1°C and humidity of 55 ± 10%, and allowed free access to food and water in a 12-hour light/dark cycle. | MWM, Golgi staining | [57] |
| 60 SAMP8 | Manipulation methods for 30 seconds at each point. | 6 days a week for 4 weeks | Housed at 25°C, and allowed free access to food and water. | MWM, Immunofluorescence staining, Detection of differentially expressed genes, iTRAQ | [58] |
| 48 Wistar | / | Once a day for 15 days (no treatment on the eighth day) | Housed at 24 ± 2°C, and allowed free access to food and water in a 12-hour light/dark cycle. | MWM, Electrophysiological experiments, LTP and LTD recording | [61] |
| 48 SD | / | 6 days | According to the Guideline for Use and Care of Medical Laboratory Animals from the Ministry of Public Health of People’s Republic of China. | Behavior deficit score, Sensory test, IHC Staining, Electrophysiology | [62] |
| 48 SD | / | 30 days | According to the Guideline for Use and Care of Medical Laboratory Animals from the Ministry of Public Health of People’s Republic of China. | MWM, Navigation test, Spatial probe test, Western Blots | [63] |
| 30 (20 APP/PS1 and 10 C57BL/6) | / | Once every other day for 4 weeks | Housed at 23 ± 2°C and humidity of 40 - 60%, and allowed free access to food and water. | MWM, IHC and immunofluorescence staining, Western Blots | [64] |
| NA | / | 30 days | According to the Guideline for Use and Care of Medical Laboratory Animals from the Ministry of Public Health of People’s Republic of China and the National Institute of Health Guide for the Care and Use of Laboratory Animals (NIH Publications No. 80-23) | Passive avoidance test, Active avoidance test, MWM, Electrophysiology | [65] |
| 24 Wistar | 1 min/day | 14 days (1day rest after 6 days treatment) | According to the Guideline for Use and Care of Medical Laboratory Animals from the Ministry of Public Health of People’s Republic of China. | MWM, Western Blots, Electrophysiology, iTRAQ, nissl staining and DHE staining, Microinjection into intracerebroventricular | [66] |
| 240 Wistar | 1 min/day | 14 days | NA | MWM, Microdialysis, Immunofluorescence staining, Electrophysiology | [67] |
| NA | NA | 14 days (1day rest after 6 days treatment) | Housed at 22 ± 2°C and humidity of 55 ± 10%, and allowed free access to food and water in a 12-hour light/dark cycle. | Western Blots, ELISA, Immunofluorescence staining, Electrophysiology, RT-PCR | [68] |
| NA | 10 min/day | 14 days (1day rest after 6 days treatment) | According to the National Institute of Health Guide for the Care and Use of Laboratory Animals (NIH Publications No. 80-23). | NOR, Radial Arm Maze, Western Blots, ELISA, electrophysiology, CSF collection, Plasma collection | [69] |
| 30 SD | / | 7 days | NA | Step-down test, LTP recording of hippocampal CA1 area | [70] |
| 18 (12 APP/PS1 and 6 C57BL/6) | / | 3 times per week for 6 weeks | According to the Guideline for Use and Care of Medical Laboratory Animals from the Ministry of Public Health of People’s Republic of China. | IHC and Immunofluorescence staining, Western blots | [75] |
| 63 (42 SAMP8 and 21 SAMR1) | / | 8 days, and 2 days of rest, for a period of 30 days | Housed at constant temperature, and allowed free access to food and water in a 12-hour light/dark cycle. | TEM, IHC staining, Western blots, RT-PCR | [76] |
| 115 Wistar | / | Once a day for 15 days (no treatment on the eighth day) | According to the Guiding Recommendation for the Care of Laboratory Animals issued by the Ministry of Science and Technology of the People's Republic of China in 2006. | MWM, IHC staining, Western blots | [77] |
| 20 Wistar | / | once | According to the Guideline for Use and Care of Medical Laboratory Animals from the Ministry of Public Health of People’s Republic of China. | electrophysiology | [78] |
| 33 SD | NA | 14 days | According to the National Institute of Health Guide for the Care and Use of Laboratory Animals (NIH Publications No. 80-23). | MWM, Passive avoidance test, Open field test, IHC staining, RT-PCR | [81] |
| 60 SD | / | 7 days | Housed under pathogen-free conditions, and allowed free access to food and water in a 12-hour light/dark cycle. | MWM, Immunofluorescence staining, Western blots, 2,3,5-TTC staining, ELISA | [86] |
| NA | / | 28 days with a rest every 7 days | NA | MWM, Immunofluorescence and Hoechst 33342 staining, Western Blots, RT-PCR | [89] |
| 30 APP/PS1 | / | 4 weeks | According to the National Institute of Health Guide for the Care and Use of Laboratory Animals (NIH Publications No. 80-23). | MWM, TUNEL staining, IHC staining, Western blots | [96] |
| 48 APP/PS1 | / | 4 weeks | Housed at 21 - 25°C and humidity of 60 - 70%, and allowed free access to food and water. | MWM, step-down test, Magnetic resonance Imaging, Magnetic Resonance spectroscopy, Nissl staining, Western blots | [97] |
| 72 | Manipulation methods, each acupuncture session lasted 3-4 hours. | 6 times a week, over the course of 8 weeks | / | MMSE, LOTCA, FMA, mADL, ELISA | [98] |
| 50 (40 SAMP8 and 10 SAMR1) | Needles were rotated at the rate of twice per second for 30 seconds at each point. | 15 days (no treatment on 8th day) | According to the National Institute of Health Guide for the Care and Use of Laboratory Animals (NIH Publications No. 80-23). | MWM, micro-PET, H&E staining, IHC staining | [101] |
| 24 SD | / | 30 days | NA | MWM, navigation test, spatial probe test, Western Blots | [109] |

Supplementary Table 2 Characteristics of included human studies.

| Sample size | Stimulation parameter | Treatment course | Assessment of experimental outcomes | Refs. |
| --- | --- | --- | --- | --- |
|  |  |  |  |  |
|  |  |  |  |  |
| 28 (14 AD and 14 HCs) | 3 mins | Once | MMSE, MoCA, RS-fMRI | [23, 117] |
| 36 (8 MCI, 14 AD, and 14 HCs) | 3 mins | Once | MMSE, AVLT, RS-FMRI | [113] |
| 49 (14 MCI, 21 AD, and 14 HCs) | 3 mins | Once | MMSE, AVLT, CDR, RS-FMRI | [118] |
| 24 (12 MCI and 12 HCs) | needle was inserted from the beginning, and after resting for 1 min, the needle was manipulated for 2 mins. | Once | MMSE, CDR, RS-FMRI | [119-121] |
| 12 (6 AD and 6 HCs) | / | Once | RS-fMRI | [122] |
| 26 AD | NA | NA | RS-fMRI | [123] |
| 32 MCI | each needle was manually twirled at an angle of ± 60° and a rate of approximately 120 times per minute. | 5 times a week, on weekdays, for 4 weeks | MoCA, digit-symbol substitution test, digit-span test, ADAS-Cog, RS-fMRI | [124] |
| 78 (28 MCI and 50 HCs) | Each needle twirled using mild reinforcing-reducing method. The needles would be retained for 20 minutes and be rotated 5 times. | 3 times a week, 4 weeks as a course. The interval between the two courses was 2 weeks of rest, with a total of 4 courses in 6 months. | MMSE, MoCA, AVLT, CDR, RS-FMRI | [125] |

Supplementary Table 3 List of abbreviations.

**Abbreviations**

| Alzheimer’s disease | AD |
| --- | --- |
| amnestic mild cognitive impairment | aMCI |
| AMP-activated protein kinase | AMPK |
| amyloid beta | Aβ |
| acetylcholine transporter | AChT |
| amyloid-protein precursor /γ-secretase | APP/PS1 |
| adrenergic receptor | AR |
| Animal Research: Reporting In Vivo Experiments Guidelines | ARRIVE |
| Alzheimer's Disease Assessment Scale-Cognitive Subscale | ADAS-Cog |
| Auditory Verbal Learning Test | AVLT |
| β-Site amyloid precursor protein cleaving enzyme 1 | BACE1 |
| Bcl-2-associated X protein | Bax |
| bilateral common carotid artery stenosis | BCAS |
| B-cell lymphoma-2 | Bcl-2 |
| brain-derived neurotrophic factor | BDNF |
| brodmann areas | BA |
| Bromodeoxyuridine | BrdU |
| cAMP-response element-binding protein | CREB |
| Cerebrospinal Fluid | CSF |
| choline acetyltransferase | ChAT |
| Clinical Dementia Rate | CDR |
| corpus callosum | CC |
| choline transporter 1 | CHT1 |
| cingulate gyrus | CG |
| dopamine | D |
| dopamine- β-hydroxylase | DBH |
| dentate gyrus | DG |
| dihydroethidium | DHE |
| default mode network | DMN |
| electroacupuncture | EA |
| Enzyme-linked immunosorbent assay | ELISA |
| epidermal growth factor | EGF |
| eukaryotic elongation factor-2 kinase | eEF2K |
| eukaryotic translation initiation factor 4E | eIF4E |
| eukaryotic Y-box-binding protein | YB-1 |
| excitatory postsynaptic potential | EPSP |
| fibroblast growth factor | FGF |
| Fugl-Meyer assessment | FMA |
| five familial mutation | 5XFAD |
| γ-aminobutyric acid | GABA |
| growth associated protein | GAP |
| glial cell line-derived neurotrophic factor | GDNF |
| glial fibrillary acidic protein | GFAP |
| glutamate | Glu |
| glutamate receptors | GluRs |
| glycogen synthase kinase 3 beta | GSK3β |
| hairy and enhancer of split 1 | Hes1 |
| healthy controls | HCs |
| hematoxylin & eosin | H&E |
| immunohistochemistry | IHC |
| inferior parietal lobule | IPL |
| inferior temporal gyrus | ITG |
| isobaric tags for relative and absolute quantitation | iTRAQ |
| Loewenstein Occupational Therapy Cognition Assessment | LOTCA |
| long-term depression | LTD |
| long-term potentiation | LTP |
| microglia | MG |
| myoinositol | mI |
| manual acupuncture | MA |
| middle cerebral artery occlusion | MCAO |
| mild cognitive impairment | MCI |
| Mini Mental State Examination | MMSE |
| modified Activity of Daily Living | mADL |
| modified neurological severity scores | mNSS |
| Montreal Cognitive Assessment | MoCA |
| middle temporal gyrus | MTG |
| mammalian target of rapamycin | mTOR |
| Morris Water Maze | MWM |
| norepinephrine | NE |
| Novel object recognition | NOR |
| neurotrophin | NT |
| N-Acetylaspartate | NAA |
| neurofibrillary tangles | NFTs |
| nerve growth factor | NGF |
| neuronal stem cells | NSCs |
| neuron-specific nuclear protein | NeuN |
| N-methyl-D-aspartate receptor | NMDAR |
| N-methyl-D-aspartate receptor subtype 1 | NMDAR1 |
| oligodendrocyte precursor cells | OPCs |
| oligodendrocyte | OL |
| ostuka Long-Evans Tokushima Fatty | OLETF |
| precuneus | PCu |
| positron-emission tomography | PET |
| posterior cingulate cortex | PCC |
| post-stroke cognitive impairment | PSCI |
| postsynaptic density 95 | PSD-95 |
| protein kinase A | PKA |
| P38 mitogen-activated protein kinases | P38MAPK |
| p75 neurotrophin receptor | p75NTR |
| precursor of BDNF | proBDNF |
| p70 ribosomal protein S6 | p70S6 |
| population spikes | PS |
| right middle temporal gyrus | MTG |
| reactive oxygen species | ROS |
| resting state functional magnetic resonance imaging | RS-fMRI |
| Reverse Transcription-polymerase Chain Reaction | RT-PCR |
| scopolamine | SCO |
| Sprague-Dawley | SD |
| synaptophysin | SYN |
| senescence-accelerated mouse prone | SAMP |
| senescence-resistant inbred strains | SAMR |
| subgranular zone | SGZ |
| subventricular zone | SVZ |
| Transmission electron microscopy | TEM |
| triphenyl tetrazolium chloride | TTC |
| Terminal deoxynucleotidyl transferase dUTP nick end labeling | TUNEL |
| two-vessel occlusion | 2VO |
| transient receptor potential vanilloid subtype 1 | TRPV1 |
| tyrosine receptor kinase B | Trk-B |
| vesicular acetylcholine transporter | VAChT |
| vascular cognitive impairment | VCI |
| vascular dementia | VaD |
| vascular endothelial growth factor | VEGF |
| ventricular zone | VZ |
